# Purple: A Modular System for Developing and Deploying Behavioral Intervention Technologies

**DOI:** 10.2196/jmir.3376

**Published:** 2014-07-30

**Authors:** Stephen M Schueller, Mark Begale, Frank J Penedo, David C Mohr

**Affiliations:** ^1^Center for Behavioral Intervention TechnologiesDepartment of Preventive MedicineNorthwestern UniversityChicago, ILUnited States; ^2^Northwestern UniversityDepartment of Medical Social SciencesChicago, ILUnited States

**Keywords:** software tools, software engineering, open source, evaluation methodology, Internet intervention, mobile intervention, mobile health

## Abstract

The creation, deployment, and evaluation of Web-based and mobile-based applications for health, mental health, and wellness within research settings has tended to be siloed, with each research group developing their own systems and features. This has led to technological features and products that are not sharable across research teams, thereby limiting collaboration, reducing the speed of dissemination, and raising the bar for entry into this area of research. This paper provides an overview of Purple, an extensible, modular, and repurposable system created for the development of Web-based and mobile-based applications for health behavior change. Purple contains features required to construct applications and to manage and evaluate research trials using these applications. Core functionality of Purple includes elements that support user management, content authorship, content delivery, and data management. We discuss the history and development of the Purple system guided by the rationale of producing a system that allows greater collaboration and understanding across research teams interested in investigating similar questions and using similar methods. Purple provides a useful tool to meet the needs of stakeholders involved in the creation, provision, and usage of eHealth and mHealth applications. Housed in a non-profit, academic institution, Purple also offers the potential to facilitate the diffusion of knowledge across the research community and improve our capacity to deliver useful and usable applications that support the behavior change of end users.

## Introduction

Health care stakeholders are increasingly turning to information and communication technologies to support the development and deployment of interventions aimed at supporting behavior change related to health, mental health, and wellness. These interventions, which we refer to as “behavioral intervention technologies” (BITs) [1,2], make use of Internet-connected devices, such as desktop computers, mobile devices, and/or sensors to provide low-cost, scalable, and effective methods to expand the portfolio of tools available and to enhance the reach, impact, and speedy implementation of current research and practice [3,4]. While the literature on the clinical application of BITs has been growing at an enormous rate, much of the discussion on development methods has been confined to the engineering and computer science communities through conference proceedings of meetings such as CHI (Computer-Human Interaction), UbiComp (Ubiquitous Computing), Pervasive Health, and MobiSys (Mobile Systems). BIT development involves considerable challenges [2], not the least of which stems from the necessary collaboration between the clinical researchers, who most often develop the idea for the intervention, and technologists who subsequently create the BIT. Despite the growing number of research teams and resultant BITs, most efforts in this space lack a generalizable technological approach to their development, often producing divergent platforms and software to meet similar needs, solve similar problems, and create BITs with similar sets of features. This paper, intended for a broad readership, describes Purple, a modular system that supports the development and deployment of BITs.

Purple is an iteratively constructed system that has evolved to address the needs of stakeholders who create, deploy, and use BITs, including researchers, technologists, clinicians, and patients. We begin with an overview of Purple’s underlying rationale and history. Because the needs of stakeholders guided the development of Purple’s capabilities, we present a brief description of BIT stakeholders as they relate to Purple. We then provide a brief review of Purple’s current technical features. To give the reader a more concrete understanding of these features, we present two case studies of BITs developed using Purple. The diversity of these case studies in clinical approach and technological componentry illustrates the flexibility of Purple. Yet the use of generalizable components illustrates the usefulness of taking a modular approach with an eye towards repurposing components. This paper aims to aid stakeholders interested in this space by outlining the necessary components of a BIT development system and presenting the features of Purple that seek to address each of these needs.

## Purple

### Overview

The field of digital health generally, and BIT research specifically, is an emerging area of transdisciplinary research that requires specific expertise from multiple disciplines as well as the development of shared conceptual frameworks that transcend the perspectives of any one field [5]. Purple aims to address these needs by providing a framework that supports the development of BITs in a manner that is, as much as possible, extensible, modular, and repurposable for a community of researchers. This framework allows individual stakeholders to make use of the contributions and development provided by others and contribute based on their specific expertise.

Many BITs, even when targeting very different behaviors and clinical outcomes, require similar features. These features include methods of enrolling users, user authentication, and content delivery, and the ability to receive, store, and manipulate user information and feed it back to users and investigators on a variety of devices (eg, mobile phones, computers, and tablets). Currently, individual research labs that develop BITs as one-off projects often recreate these features repeatedly. Although some labs have the capacity to build modular systems and repurpose code for subsequent projects, this is the exception rather than the rule with regards to development related to clinical research. Thus, one goal of Purple was to build a system that leverages resources across multiple researchers, labs, and institutions. Indeed, other resources exist that target some of the same goals of Purple, including Open mHealth [6], LifeGuide [7], and the Behavior Wizard [7]. These include acting as a repository of developed code for mHealth projects [6], providing conceptual guidance [8], and reducing the need for programming knowledge via open-source software that allows the creation and modification of BITs using authoring tools and logic commands [7]. Purple arose concurrently with these resources. However, as none provided a sufficient solution for the problems encountered by clinical researchers (eg, eliminating the need for a programmer) or sufficiently covered the user management, recruitment, and data acquisition needs for our various projects, we developed Purple to facilitate the development of our own projects as well as external ones.

We first created generalizable features that addressed the required elements for any BIT (eg, user management, recruitment, and data acquisition) and the capabilities to support the development of new features, which over time can be formed into generalizable features, and then be made available to researchers. Developing each of these features could take anywhere between a few days to a few weeks of developer time depending on whether a similar feature (ie, with existing available code) developed for another need (eg, a news site or blog editor) could be sufficiently modified or if the required feature needs to be constructed from scratch. Through sharing and iteration of features and the knowledge associated with developing, deploying, and evaluating them, Purple provides avenues for the dissemination of technical and design information outside of traditional academic papers and conferences. Table 1 displays a list of currently available technological features.

**Table 1:** Technical features of Purple.

Features			Creators	End users	Essential	Optional
**User management**						
	**User authentication**					
		Study IDs, passwords	X	X	X	
		Password generation	X	X	X	
	**User definition**					
		Personal health information management	X	X	X	
	**Study management**					
		Study/intervention assignment, randomization	X		X	
**Content authorship/management**						
	**Didactic content**					
		Content editor	X			X
		Generic form builder	X			X
		Content type (eg, text, images, video, audio)	X	X		X
		Progression (eg, sequential, non-sequential)	X		X	
	**Assessments**					
		Self-report types (checkbox, slider, text, etc)		X		X
		Phone sensor acquisition		X		X
		External data API access		X		X
		Intra-assessment logic (sequential, adaptive, etc)	X		X	
		Scoring (summing, complex algorithms)	X	X	X	
	**BIT construction**					
		Progression rule definition (eg, chronological, task-based, time-based, custom)	X	X	X	
		Custom interaction tool authorship	X			X
**Content delivery**						
		Platform (phone, tablet, Web, etc)	X	X	X	
		Connectivity (online, offline, both)	X	X	X	
		Interfaces (touch, mouse, keyboard, etc)	X	X	X	
**Data management**						
		Access	X		X	
		Visualization (charting, custom, InfoVis)	X	X		X

The use of Purple involves processes that have been and continue to be developed to facilitate collaboration between clinical investigators and technologists across the life of a project [5,9,10]. Because BIT development commonly uses iterative development processes, new features are developed with the maximum degree of flexibility possible to allow investigators to adapt the characteristics of those features over the development process. The need for the Purple architecture was born out of our experience. Like many investigators, we began work using a commercial developer who built a website that met our specifications for an Internet intervention for depression [11]. Through evaluation, we encountered many flaws in our design and conceptualization, we developed new ideas regarding methods of engaging users, and we found colleagues who wanted to build on our work. Unfortunately, the one-off construction we used limited our ability to adapt, improve, and share our work. Our next project, an intervention for cancer survivors called Onward [12], employed a development strategy that prioritized modularity and flexibility. The features developed within Onward became the beginning of the feature set available in Purple. Features from Onward are now used to further development on Onward-like interventions and have been adapted for several Web-based and tablet interventions targeting a variety of clinical problems, such as PC CHIP, a tablet-based intervention described below. Similarly, our work in mobile interventions began with the retooling of Mobilyze, an intervention for depression built for Nokia’s Symbian operating system [13] for the Android platform. This was done concurrently with work on FOCUS, a smartphone intervention for schizophrenia [14]. The work completed on these two projects created an infrastructure of mobile features for Purple that is now being used by multiple projects. Initial efforts in the creation of Web and mobile features developed within the modular Purple framework were given a significant boost in 2011, with the establishment of the Center for Behavioral Intervention Technologies (CBITs), with support from the National Institute of Mental Health and additional funding from Northwestern University.

With the establishment of CBITs, we began to expand our services, and by extension the community of researchers contributing to and deriving benefit from Purple, to investigators outside of Northwestern University. As of this writing, CBITs supports more than 50 projects in the United States, Africa, and South America, funded by more than 20 National Institutes of Health (NIH) grants, 2 grants from the Canadian government, and numerous other government agencies, foundations, and universities. The expansion of research teams using and contributing to Purple helps achieve its overarching vision to become a hub for the community of BIT researchers that allows for the transfer of development methods via recycling of code across research labs, allowing the researchers to rapidly benefit from the knowledge gained by the larger community of researchers, as well as the larger group of stakeholders.

Another aspirational vision for Purple is to develop databases that will allow the community of researchers to answer questions that no individual lab can address in isolation. For example, lab-based research allows investigators to examine how specific features or characteristics are related to adherence and outcomes within their own intervention. These data can be examined across research using meta-analytic techniques (eg, [15]); however, as meta-analyses combine only aggregated data, they have limited power to detect effects and can only address questions at a gross level, using the kinds of information that are collected routinely across research times and reported in publications. In cases where individual subject data are available, the data can be pooled to conduct meta-analyses on the original data, also known as mega-analysis [16]. Mega-analyses are preferable to meta-analyses when only a few relevant studies exist or subgroup analyses are desired. Both of these are often true in the domain of BITs. The speed of technological innovation means researchers must base decisions on conclusions drawn from few, and often outdated, studies. Furthermore, researchers are often interested in how the user groups they are targeting (eg, clinical needs, population, setting) might use and benefit from particular BITs or BIT elements. The most powerful mega-analyses would come from studies that share a common framework that guides methodological decisions (eg, delivery of treatment components, outcomes assessed). Within the Purple system, use and outcome data are consistently defined and application features and characteristics are consistently identified. While users of Purple are not required to share data, if they choose to do so, the data are added to a de-identified database. This database provides the opportunity to evaluate the relationship between specific design elements, as well as use and clinical outcomes. As this database grows, it will provide a resource to enhance the evidence base that could inform how best to design BITs for general clinical purposes, and for specific populations and settings. As it stands, several collaborators have stored their data on our servers; however, the proper ontologies still need to be created to make use of this data. As such, data have yet to be combined and analyzed cross-projects, and this remains an aspiration rather than an application.

Purple developers have made an intentional decision to maintain this system in a non-profit university setting. The primary goal is to support research rather than to develop a product. As research requires testing intervention principles, Purple is optimized for the extreme data needs of researchers, including the collection of detailed data to permit fine-grained analyses of use patterns of study participants. Furthermore, research focuses on the creation and dissemination of knowledge. As developed components are available to be independently disseminated, they are opensourced to the community at large. For example, “Purple Robot”, a real-time mobile phone sensor data acquisition platform that can collect all available data from Android phones, is currently available for download on the GooglePlay store with source code available at GitHub [17].

### Stakeholders Involved in Behavioral Intervention Technologies

**Figure 1:**
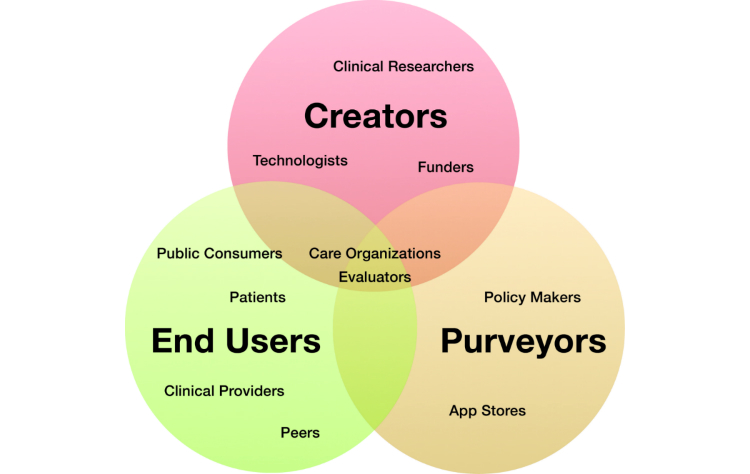
Stakeholders involved in BITs.

The creation of a development framework must consider the needs of all stakeholders, who can be involved in the design, development, funding, deployment and use of BITs. We present in Figure 1 the highest level of classes—creators, end users, and purveyors—as overlapping categories, with more specific stakeholders selected who may play multiple roles within the process of developing and deploying BITs, represented within these categories [18]. The creator class encompasses all stakeholders who construct, shape, improve, and disseminate a behavioral intervention technology and can be grouped into subclasses such as clinical researchers, technologists, and funders. End users make use of the content and resources provided by BIT creators. They are the ones who actually engage with the intervention in order to facilitate the behavior changes desired by the creators. End users may include patients or public consumers, clinical providers who interface with the applications, or peers who are networked through the application. End users also author the data necessary to evaluate the efficacy of the BIT at reaching its stated goal. This includes both use data that correspond to an end user’s engagement with the BIT and clinical data that correspond to the specific clinical and behavior change targets. Purveyors are those responsible for the dissemination the intervention and may include app stores or care providing organizations, as well as policy makers. Many stakeholders may have roles in several classes, which is why the highest categories are presented as overlapping within Figure 1. For example, clinical care organizations are commonly purveyors but may also be involved in the creation and the use of interventions.

Although features of Purple have been developed with most of these stakeholders in mind, for the purposes of the current paper we focus most directly on creators, who use Purple to instantiate their intentions and aims into code and produce a BIT, and end users whose behavior the BIT is intended to support or change.

### Technical Features

#### Overview

Over time, Purple has expanded to include a range of technical features. Table 1 displays many of these features and groups them into four categories representing core functionality: user management, content authorship, content delivery, and data management. The table includes an indication of the class of stakeholders each feature is aimed at and whether that feature is essential or optional to the functioning of a BIT in the Purple System. “Essential” features are those that are necessary to deploy and evaluate a BIT in a research context and thus appear in every BIT developed using Purple (eg, user management, recruitment/enrollment). These features have been developed to be generalizable across projects because of their recurring need in each. Optional features are unique to a given project and created in response to specifications of the producers for that BIT. These features are typically developed as non-generalizable features that over time might be able to be modified for use across projects. Last, Purple contains many generalizable but optional features. Generalizable but optional features are created when the demand across projects is such that it makes sense to create a generalizable solution like a dashboard to allow a clinician or coach to view data and manage BIT users (eg, [19]) or through the development of an initially non-generalizable feature into a generalizable one. This table provides a menu of options of the current capacities of Purple, but new capacities are frequently developed in response to new problem spaces or through new collaborations. We discuss each of these feature groups in more detail below.

#### User Management

User management is an important element for any BIT to ensure that data are structured and interpretable to allow for evaluation. As such, the BIT system must track who enters the system, when, and be able to provide access to the correct information and content for each individual user and stakeholder. This is also crucial for practice to ensure privacy and security concerns, promote trust in the BIT [20,21], and support user experience by ensuring that it works properly when content is tailored or personalized.

To support privacy and security, an essential feature within Purple is to allow users to create unique identifiers, passwords, and to assign these users to a specific study. In Purple, these processes make up user authentication features. Another aspect of user management is defining variables related to that user and specifying how these variables correspond to other features within the intervention. For example, Purple user management includes the ability to define which stakeholders are permitted access to demographic or personal health information (PHI). Some instances of PHI stored within Purple should not be viewable by most personnel and stakeholders. For instance, email addresses or phone numbers of end users may be required for Purple to deliver automated email or text messages; however, most people engaging with or receiving data from Purple do not require access to such information and therefore should not see it. User authentication also involves validating the user’s credential against different features of the intervention including the date the intervention started, condition membership (eg, experimental vs control condition), and other user specific permissions. This allows the intervention provided to each user to differ according to conditions preset by the creators.

#### Content Authorship

Content authorship tools enable creators with limited technological sophistication to participate more directly and easily in the development process. This is useful for creating BITs, as experts in behavior change often do not have the capability to program BITs on their own and need to team with technologists who do. We use the term content broadly to refer to anything that is provided to a user (eg, text, video, or audio) for didactic purposes; for example, text “lessons” that are commonly used in BITs such as Internet sites or mobile applications [13,22], assessments, notifications (email, secure messaging, in-phone notifications), or feedback. Although many behavioral scientists might see these as functionally different, we discuss them together because, from the vantage of the Purple system, they use similar or identical technical features. For example, the primary difference between a piece of didactic content and an assessment within Purple is that the didactic content only displays text, images, video and/or audio, while assessment combines this with another element that allows the user to input information.

Purple contains several content authorship tools to meet the needs of the creator. These can be generally classified as tools to create didactic content, assessments, and BIT construction. As much as possible, these tools are designed so that individuals without programming knowledge can create basic didactic content, notifications, or modify elements of interactive tools to meet the more specific requirements of the application. The content editor allows the creator to enter text and upload images, video, and audio, which will then be displayed back to the end user. The editor is useful at creating BITs that draw on specific types of scripted interactions (eg, a user progressing through a series of slides). As a majority of BITs use similar types of interactions, content authorship tools provide a useful low-effort way to script and pilot content and intervention ideas before progressing to more advanced programming required in more complicated and BIT-specific interaction flows. We use examples below to demonstrate how content authorship tools are used in two different BITs, but across projects these tools are ones that empower clinical researchers to create and iterate aspects of the intervention.

Assessment refers to the acquisition and scoring of data. The content editor also allows the creator to input the content of questions for self-report data and to determine the presentation of those questions, such as the number of questions on a page and the type of response required, such as a check box, slider, or free text. A variety of intra-assessment logic types are available, including simple logic such as sequential or random presentation of assessment items, adaptive presentation making use of Boolean logic such as if-then or more complex logic, or item-response theory. Purple also has the capacity to pipe responses from one question into the text of another question.

Purple allows for the collection of a wide variety of additional data important for use and usability evaluation, as well as ubiquitous computing. Within Purple, detail log and use data are collected from sensors and applications running on a website or mobile device, as well as data from other sources via application programming interfaces (APIs). Purple Robot [17] is a mobile application within the stack of Purple applications that supports the acquisition of a full range of mobile phone sensors (eg, global positioning system [GPS], Wi-Fi, accelerometers, Bluetooth, altimeters, light sensors), device information (eg, battery, running software, and apps), and options to scan for visible external devices (eg, Bluetooth devices or wireless access points). Purple Robot also provides a JavaScript API that has been used to link external devices (eg, Fitbit, Jawbone, wireless pillboxes) and allows for additional sensors to be added in the future. In order to facilitate the transfer of data into meaningful information, Purple offers a variety of scoring options. These range from simple summation to more complex algorithms and machine learning functions.

These fundamental tools for the creation of didactic content, assessment, and logic can be combined to create interactive tools. Interactive tools developed so far using Purple include goal-setting tools, activity scheduling and monitoring tools (using calendars), and tools that promote thought restructuring. These elements can also be modified and combined in new ways to create custom interaction tools. For example, altering a tool from tracking food to tracking activity can alter an intervention from targeting diet for weight loss to behavioral activation for depression. A generic form builder within Purple creates many of these tools. The generic form builder allows a creator to provide free text boxes or other modes of data entry and then to provide this information back to user at specified times. The generic form builder is also able to repurpose code for different modes of entry (such as on a calendar or on a feed) that have been developed for various projects within Purple.

Progression rules refer to the rules that define how a user moves through an intervention. While some BITs allow access to all elements of an intervention from the first login, others contain rules that sequence the presentation of treatment elements. While complex progression rules can require programming effort, the editor also contains features that allow creators to set basic progression rules. For example, the creator may specify that lesson, tool, or notification should be available to the end user after performing specific tasks, reaching or not reaching a treatment goal, or simply the passage of a specific amount of time. Creators can also define within each lesson when specific pages should be available to end users. This includes sequential logic definitions (eg, page 2 displays after page 1) or non-sequential logic (page 3 displays only after a certain condition is met, such as number of views or score on an assessment). These tools can also be applied to tailoring interventions to user characteristics.

#### Content Delivery

In the context of BITs, treatment elements can be delivered in multiple modalities to multiple users (eg, patients/consumers, coaches, clinicians) and according to previously specified progression rules (as discussed above). Some intervention delivery methods have been requested and used in so many of the BITs designed within Purple that features have been developed specifically for those purposes. These include calendar displays and graphing tools. Other content varies significantly from BIT to BIT but requires the researcher to make specific decisions to shape the end user’s experience.

Content delivery essentially enables the intervention to be played to the end user on their device or computer based on rules defined in the content authorship and management elements in Purple. Content delivery in the Purple system uses HTML5-based hybrid applications. Hybrid applications allow for the production of a current generation, Web-based experience for end users without writing specific Android or iOS code. For user-facing portions, these applications can be deployed to Android, iOS, or Windows mobile devices. This allows for deployment on multiple platforms. Designing for multiple platforms (or optimizing for a specific platform) involves several decisions that require an understanding of the desired user interaction. Some of our applications are completely on the client side (eg, they make use of the browser side and do not require an active Internet connection), whereas others are server side, thus requiring an active connection. In order to reuse the code developed for online applications, we use Adobe PhoneGap/Apache Cordova software to package the application so they work offline, allowing creators to deliver interventions that do not necessarily require connectivity. The use of HTML-5 hybrid development also supports the more rapid production of prototypes, facilitates testing directly on desktop or laptop computers, and uses a more generally available programming skillset.

In addition, by using HTML5-based hybrid applications, the user interface can be adapted to the delivery platform. For example, a traditional check box is easy to select with mouse-driven interfaces. The same box would be encased in a button for touch screen devices to simplify user selection. Thus, multiple versions of tools are created, allowing the user experience to be tailored for mobile phones, tablets, and desktop computers. Important human-computer interaction issues here might include making specific interface-related decisions, for example, using a library to make Web applications more responsive on the phone or determining what the concept of a “hover” action might be when it does not exist on a tablet but does on a desktop computer. Purple offers a system where many of these design possibilities are mapped out, allowing the designer to make decisions based on the intervention concept and needs of the target population.

#### Data Management

Data are used for a variety of purposes, including the evaluation of the BIT, as well as to manage and define the user experience. In general, two types of outcomes are of interest to researchers evaluating BITs: clinical outcomes and use (or adherence) outcomes. Clinical outcomes represent the treatment target, such as weight, smoking cessation, or depression. These outcomes typically use participant self-reports, or observer reports (eg, clinicians) collected via assessments conducted within the system but may also use project specific APIs or sensors. Use data can be measured in a variety of ways including the frequency that a participant has logged in or launched a BIT, the features of the BIT used, and the length of time interacting with the BIT. While Purple has the capacity to collect fine grained data of every interaction, it is usually preferable to pre-specify the use data desired in advance because collection of all available data leads to significant demands on the server and provides so much data that it is difficult to sort through, understand, analyze, and draw conclusions. Data can be made available to researchers in raw data dumps in commonly used formats such as Excel, or through direct access to databases to facilitate real-time data analysis.

End users, and sometimes researchers, often require data to be visualized to facilitate understanding. A variety of charting libraries are available that can be used to create traditional bar, line, and pie charts. Purple also allows for the development of custom authored visualizations that allow creators to develop creative methods of displaying data for specific use cases. InfoVis combines charting or custom visualizations with didactic content to create visual representations that can be effectively deployed to enhance understanding.

Purple contains sets of functionality that map onto common use cases. Novel configurations will require programmer effort until these become sufficiently standardized to allow the creation of modular elements. Within and between the BIT elements, there is a “handshake” between features that requires technological developer support. As much as possible, the editor allows non-programmer creators to develop and import content, manage assessments, define progression rules, and access data, maximizing control and management of the development process in the hands of clinical researchers. In almost all cases, we use structured query language (SQL)–backed data storage, which most modern developers can use to interface with the data directly. Some projects using Purple house data on our servers, whereas other collaborators store data on their own server.

### Case Studies

#### Overview

The modular structure of the Purple system allows common technical features within Purple to be integrated to create unique BITs. We present here two examples of BITs developed using the Purple system, which have different clinical aims, target populations, and use different devices, to illustrate how similar Purple features can be used to create very different interventions. Table 2 displays brief descriptions of manifestations of the technical features in Purple across these two interventions. A more detailed description of each intervention follows.

**Table 2:** Technical features within intervention case studies.

Features			Mobilyze	PC CHIP
**User management**				
	**User authentication**			
		Study IDs, passwords	Performed by creator at randomization	Performed by creator at start of project
		Password generation	None	Single password set for all users
	**User definition**			
		Personal health information management	Email (within Purple Robot)	Age, gender, email (for contact)
	**Study management**			
		Study/intervention assignment, randomization	Content released daily	Content unlocked weekly, questions released 1 hour after scheduled session, user specific permissions and group memberships
**Content authorship/management**				
	**Didactic content**			
		Content editor	Used to enter and schedule release of text lessons	Used to enter text, video, and audio
		Generic form builder	Calendar tool, active coping tool, coping card	External tools used through assessment center API
		Content type (eg, text, images, video, audio)	Text	Text, video, audio
		Progression (eg, sequential, non-sequential)	Sequential; new lessons available daily	Sequential; new content unlocks weekly
	**Assessments**			
		Self-report types (checkbox, slider, text, etc)	All form types	Radio buttons only
		Phone sensor acquisition	Various through Purple Robot	None
		External data API access	None	WebEX (video conferencing)
		Intra-assessment logic (sequential, adaptive, etc)	Basic (lists of questions) and advanced (scoring controls progression)	Computerized adaptive testing
		Scoring (summing, complex algorithms)	Scoring occurs based on machine learning rules	Scoring uses Item-Response Theory (IRT) algorithms from Patient-Reported-Outcomes Measurement Information System (PROMIS)
	**BIT construction**			
		Progression rule definition (eg, chronological, task-based, time-based, custom)	Time-based	Time-based, linked to video conferencing session
		Custom interaction tool authorship	Form-based tools can be authored using “tool builder”	Not applicable
**Content delivery**				
		Platform (phone, tablet, Web, etc)	Smartphone	Tablet
		Connectivity (online, offline, both)	Both	Both
		Interfaces (touch, mouse, keyboard, etc)	Touch	Touch
**Data management**				
		Access	Access to data is provided about sensors using a server side database, user tool data are stored separate from sensor data	User tool usage data are accessible on the server, survey data available independently through PROMIS
		Visualization (charting, custom, InfoVis)	Graphs, list reviews, bubble visualizations	None

#### Mobilyze: A Smartphone-Based Intervention for Individuals With Depression

Mobilyze is an Android smartphone intervention based on the principles of behavior activation, which aims to reduce depressive symptoms by increasing the user’s engagement in activities that are pleasurable or provide a sense of accomplishment. The ultimate goal of the Mobilyze project is to develop a context-sensing system that harnesses data from sensors embedded within the smartphone to identify user states that may be relevant to treatment, such as location, activity, social context, and mood. As such, Mobilyze consists of three major components: (1) a patient-facing application that includes lessons and application specific tools, (2) a smartphone application that collects sensor data, monitors authored models, and provides phone interactions that support context-sensing functions, and (3) a server side framework to collect unstructured and structured user data. A pilot version of Mobilyze was evaluated with 8 end users with major depressive disorder and provided promising support for this intervention model and the development of the context-sensing system [13]. Our current work on this project involves retooling the intervention for modern platforms (ie, Android, iOS) and improving functionality related to context-sensing capabilities. This updated version of Mobilyze is a 12-week intervention consisting of content and tools consistent with principles of behavioral activation for depression [23]. Mobilyze tools include daily lessons, activity tracker, mood check-in, active coping tool, coping cards, and progress graphs.

When Mobilyze is installed on a user’s smartphone device, the end user does not have to enter any passwords or login credentials to open the application. The application does, however, need to be able to identify the user such that data entered will be linked to that user. This is accomplished using the email address associated with the phone collected by the Purple Robot application. A user can also go into Purple Robot and create a unique identifier that is different from their email address. For each user, creators are able to specify the date the intervention started for the user and release content daily corresponding to the number of days the user has been enrolled in the study.

Mobilyze consists of approximately 60 brief lessons, deployed every 1-2 days, that take no more than 5 minutes to read. These lessons were entered using the content editor authoring tool. Each day, lesson content is displayed as text via a widget set up on the home screen of the mobile device, and some lesson content links directly to other tools contained within the Mobilyze program. Lessons follow sequential release rules set within the editor. Each lesson is released on a specific day and then available to end users to view again throughout participation in the 12-week program. Other tools within Mobilyze make use of generalizable features developed for across-project use created using a generic form builder. These features include a calendaring tool (the Activity Tracker) and generic form-building tools (Active Coping and Coping Card supporting positive activities and management of stressful events). The activity tracker supports activity scheduling and monitoring that is part of behavioral activation, by allowing end users to report the date and time for the event and to rate how much pleasure and accomplishment they experienced during that event. Each element of this mode of entry corresponds to things present in other aspects of the Purple system (eg, pleasure and accomplishment are rated using slide bars that could be changed via the content authoring tool; date and time are entered using a date/time entry tool). The generic form builder allows for creation of structured interactions with the program. For example the coping card consists of some instructional content (entered via the content authorship tool), a text entry box, and assigning a date and time when that coping card will be sent to the end user as a notification.

Assessments within Mobilyze include both user-entered data and sensor data collected passively by Purple Robot. For user-entered data, a single question mood check-in requires users to report their mood using a 10-point slider bar that ranges from “bad” to “good”. All aspects of this check-in could be easily altered using the content authoring tools ranging from the question prompt, scale anchors, or type of entry mode (eg, slider, checkboxes, radio button, free text entry). Delivery of the check-in is controlled via the content authoring tool. In different phases of the deployment, it has been provided using time-based or context-based rules. The context sensing portion of this project aims to use sensor data collected from the phone to identify treatment-relevant user states. Raw data from the phone’s sensors (eg, GPS, call logs, accelerometer, application usage, screen state) are automatically collected with no effort required by the user. This context sensing requires the ability to download raw data from phone sensors as well as other external sources such as weather data, the ability to generate models that can use data to predict user states, and to upload those models to the phone where they can be used to detect user states in real time. These features were developed into an application, called Purple Robot [17]. Purple Robot provides a JavaScript API that collects the sensor data available via Android smartphone devices (eg, GPS, accelerometer, visible Wi-Fi/Bluetooth). The sensor data are linked with labeled data drawn from the user-entered responses to the mood prompts for user information about their mood to train models.

On the server side, the Purple system collects content authored by the end user within the application as data (eg, mood scores from the check-in, events, and associated ratings from the activity tracker) and detailed data related to each user’s interactions with the application. Data acquired through the sensors and use data are stored in separate databases as an additional precaution of data security and confidentiality. Use data includes when, what, and how a user clicks on each button within the Mobilyze application. For example, Purple tracks when users click on a lesson to open it, when they click on each button to progress to the next page within the lesson, and when they click on a tool or home screen button at the end of the lesson to move to another section of the Mobilyze program. From this, the creator knows when and how long a user is reading each lesson or interacting with each tool. This information is fed back to members of the study team. Coaches have an interface that provides use and clinical data that support brief calls focused on maintaining motivation and discussing aspects of the individual end user’s use of the application based on data entered into the program. Values entered via the various tools are fed back to the user as graphs displaying things such as mood over time or average pleasure and accomplishment in activities over time.

For Mobilyze, the existence of Purple Robot and the content management system allowed some development of features without additional programming but with additional content generation by the clinical team (eg, writing lessons, writing question prompts, providing feedback on visual interface). Other aspects, for example, the homepage of the application, the timing of assessment prompts, mapping servers to facilitate data transfer and storage, require programming. As such, Purple speeds the development of Mobilyze and provides a framework so that similar Mobilyze-like interventions can be more easily developed in the future.

#### PC CHIP: A Tablet-Based Intervention for Prostate Cancer Survivors

PC CHIP is a tablet-based intervention that aims to improve health-related quality of life and treatment-related symptom burden in men diagnosed with advanced or metastatic prostate cancer. The intervention consists of cognitive-behavioral stress management techniques that incorporate interactive didactics, role-plays, and content and emotional processing exercises. The intervention components also include relaxation training exercises, stress awareness, coping and communication skills provision, and social support with an emphasis on treatment-related side effects and interpersonal disruption [24]. PC CHIP is currently being evaluated in a randomized clinical trial comparing the tablet-based stress management intervention to a health promotion attention matched control condition. Throughout the 10-week intervention period, users receive weekly content through a mix of live video sessions led by a group facilitator, text slides, and audio recordings in a group format with 4-6 participants. In the health promotion condition, participants receive only didactics on health promotion topics such as the benefits of nutrition, physical activity, and treatment compliance, and other salient topics that offer information to the participants but do not involve any stress management or cognitive and emotional processing.

As users are enrolled, they are assigned to groups. Study IDs and passwords are generated by the creator at the start of the each of the group’s enrollment. Group passwords and logins are used rather than individual passwords and logins because content is consistent across end users in each group. Thus, an end user’s study ID and passwords serve to identify their group. Content is released weekly and also related to group-specific permissions. Questions related to the video sessions, for example, are released 1 hour after the scheduled session for that group.

**Figure 2:**
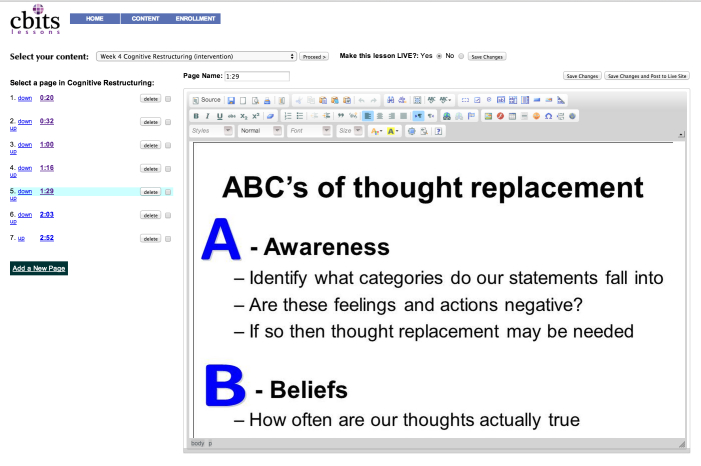
Purple “editor” content authorship and management tool.

**Figure 3:**
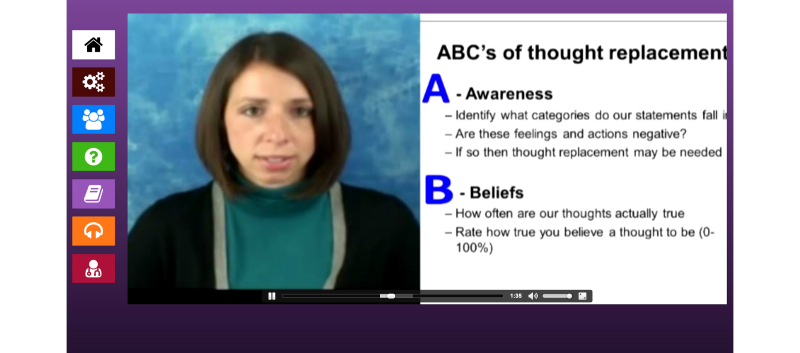
Patient-facing intervention constructed using content authorship and management tool.

PC CHIP makes use of several content authorship and management features within Purple. Purple’s content editor (displayed in Figure 2) allows entry of video content and PowerPoint slides that are then synched to each other (see Figure 3). In this way, users are able to receive content in multiple modalities at the same time. The editor is also used to upload audio files that contain the relaxation exercises and other didactics summarizing key aspects of the materials presented in the live video conferencing sessions. Progression rules within PC CHIP include both sequential and non-sequential processes. Throughout the 10-week trial, new content is available for review each week. A daily counter associates the entire intervention to a countdown clock based on a weekly video conferencing session delivered to the end users. As a result of this clock, weekly assessments and lesson content are made available directly following a weekly session.

Assessments are seamlessly provided using an external service that uses the Patient Reported Outcomes Measurement Information System (PROMIS) instruments and the Assessment Center toolkit [25]. The PROMIS Assessment Center toolkit is a free online data collection tool that allows researchers to create study-specific websites to make use of various features of the PROMIS instruments including customization of items, real-time scoring of computer adaptive tests. PROMIS instruments undergo updates and minor modifications from the PROMIS research team thus allowing researchers who use the toolkit access to the most up-to-date measures without the need to make updates themselves. Thus, connecting PC CHIP to this toolkit through Purple provides a considerable benefit with initial integration as the only programming consideration. In the PC CHIP trial, all participants are asked to log in and complete PROMIS-based computerized adaptive testing for symptoms of fatigue, pain, depression, anxiety, and physical functioning. Purple technology also provides system-generated alerts to participants with reminders to complete the PROMIS instruments. Additional API access is used to link with the WebEX video conferencing platform. WebEX is a Cisco product that supports the various needs of the PC CHIP project (ie, a single group facilitator with multiple participants). At the end of the 10 weeks, the users receive an automatically generated post-assessment evaluation that taps into the participants’ overall comfort in the sessions and perceived efficacy and understanding of the session materials. These assessments consist of radio buttons created using the Purple content authoring system.

For the PC CHIP project, Purple collects data related to use of the various features (eg, accessing WebEX, viewing presentations, accessing assessments) and the results of the post-assessment evaluation. These data are accessible on the server and provided to the creators for review of how the application is functioning, which aspects might be more beneficial, and users’ perceptions of the intervention. Outcome data are available independently through the PROMIS Assessment Center toolkit [25]. As such, no visualizations of outcome data are provided to the end users.

In the case of PC CHIP, a majority of the intervention is configured in the content authorship and management tool. With the exception of a relaxation tool that is provided only to the control group, a researcher can swap all content out with no programming knowledge. Visual elements on the home screen of the application draw content from an access and HTML database and would require knowledge of these resources and a few hours of work to change the workflow. Thus, an intervention with the exact same patient workflow could be created with no additional programming but an intervention with similar needs (eg, providing videos and PowerPoints to patients and collecting assessment data) could be constructed with a few hours of programming time.

## The Future of Purple

### Strengths and Limitations

We have presented the Purple framework and the conceptual issues related to its design and implementation as an example for the field of a modular and extensible system. The future of Purple will involve both the expansion of new features, greater translation of currently non-generalizable into generalizable features, and expansion of the research teams using and contributing to the Purple system. Although we have provided examples of BITs within Purple to show how developed features combine into a single intervention, we want to caution creators against conceptualizing and developing their interventions only at the feature-level.

One major challenge facing any development framework is to remain flexible and agile in face of the rapidly shifting technological environment. Currently, over 11,000 distinct devices exist that use the Android operating platform [26] and 23 that run iOS [27]. Furthermore, although these are currently the dominant platforms, emerging devices (eg, smartwatches, Google Glass) might significantly shift the development environment. Software updates occur even more rapidly and change the requirements and capabilities of applications running on new versions of operating systems.

Fortunately, Purple is designed to be flexible in the face of these changes. However, its ability to adapt is tied to the different contexts that participating projects provide. While investigators in the community can benefit from the investments in software and knowledge provided from those who have used Purple before them, they also contribute to the system as their projects provide new challenges, problems, and opportunities. As solutions for these problems are developed within Purple, they in turn can be passed on to the community of researchers, either through the Purple system, or when components can exist independent of the system, such as Purple Robot [17], through opensourcing to the community. As it currently stands, Purple offers a collection of features and tools (user management, content authorship, data management, and phone sensor acquisition) that do not have to be recreated by outside technologists if they want to create projects with similar aims. The future of Purple rests on its ability to guide, both conceptual and practically, the development of the next generation of BITs.

### Conclusions

The Purple system is an extensible, modular development architecture designed to support the creation of Web-based and mobile applications aimed at supporting behavior change. We have described the Purple system, focusing on the rationale guiding its development and the features available in its current state. We have demonstrated its use through a discussion of two ongoing projects investigating BITs for diverse treatment targets and populations. Last, we have presented guiding principles for the field to advance the way creators think about BITs and encourage further collaboration with the Purple framework.

While BITs hold great promise, actualizing this potential requires the capacity to transform behavior change principles into deployable interventions. Purple, now being used by more than 50 projects, facilitates this translation and does so in a way that allows for the repurposing of features and easier sharing of knowledge gained from evaluations. Purple is also a research platform that, through sharing of resources, increases capacity as well as the generalizability of knowledge.

## Abbreviations

API: application programming interfaces
BIT: behavioral intervention technology
GPS: global positioning system
NIH: National Institutes of Health
PHI: personal health information
PROMIS: patient reported outcomes measurement information system
